# Supplementary material for: MetaRibo-Seq measures translation in microbiomes
Source: Nat Commun. 2020 Jun 29;11:3268. doi: 10.1038/s41467-020-17081-z (PMC7324362; doi:10.1038/s41467-020-17081-z)
Supplement: Supplementary file 10 — Supplementary Data 7 [file 41467_2020_17081_MOESM10_ESM.zip › File2/Confidence_VeryHigh_Taxonomy/140019_out.krona.html]

Javascript must be enabled to view this page.

members
magnitude
magnitudeUnassigned
count
unassigned
taxon
rank

140019\_out

4

2
superkingdom
3

1239
3
phylum

3
class
186801

186802
3
order

541000
2
family

genus
2
216851

1
species
853

SRS020233\_contig\_number\_27693


SRS055017\_contig\_number\_36826
2292234
species
1

1
species
1897047

SRS1041031\_contig\_number\_30152


SRS1041140\_contig\_number\_13806
1
